# UV Spectrophotometric Method for Assay of the Anti-Retroviral Agent Lamivudine in Active Pharmaceutical Ingredient and in its Tablet Formulation

**DOI:** 10.4103/0975-1483.71628

**Published:** 2010

**Authors:** G Deepali, M Elvis

**Affiliations:** *Department of Pharmaceutical Chemistry, Vivekanand Education Society’s College of Pharmacy, Chembur (East), Mumbai, India*

**Keywords:** HBV, HIV, lamivudine, UV spectrophotometry

## Abstract

A rapid, simple, accurate, and economical spectrophotometric method has been developed and validated for the assay of the anti-retroviral agent lamivudine in active pharmaceutical ingredients (API) and in its tablet formulation. The analysis is based on the UV absorbance maxima at about 270nm wavelength of lamivudine, using methanol as solvent. A sample of API was dissolved in methanol to produce a solution containing 10 µg/mL of lamivudine. Similarly, a sample of ground tablets were extracted with methanol, centrifuged, and diluted with the same solvent. The absorbance of the sample preparation was measured at 270 nm against the solvent blank, and the assay was determined by comparing with the absorbance of a similarly prepared 10 µg/mL standard solution of lamivudine. The calibration graph was rectilinear from 5 µg/mL to 15 µg/mL for lamivudine with the correlation coefficient being more than 0.999. The relative standard deviation of the replicate determination was about 0.5%. The percent recovery was within the range of 98%–102%, indicating insignificant interference from the other ingredients in the formulation. The method can be applied for the routine QC quantitation of lamivudine in API and tablet formulation.

## INTRODUCTION

Lamivudine, (2R-cis)-4-amino-1-[2-(hydroxymethyl)-1,3-oxathiolan-5-yl]-2(1H)-pyrimidinone,[1] is a synthetic nucleoside analogue with activity against the human immunodeficiency virus (HIV) and hepatitis B virus (HBV).[2] The molecule has two chiral centers and is manufactured as the pure 2R, *cis*(−)-enantiomer. The racemic mixture from which lamivudine originates has antiretroviral activity but is less potent and substantially more toxic than the pure (−)-enantiomer. Compared with the (+)-enantiomer, the phosphorylated (−)-enantiomer is more resistant to cleavage from nascent RNA/DNA duplexes by cellular 3'-5' exonucleases, which may contribute to its greater potency.[3] Lamivudine is either formulated alone as a tablet/oral formulation or in combination with zidovudine. The spectroscopic method for assay of lamivudine is not official in any pharmacopoeia.[4–6] A few high-performance thin-layer chromatography (HPTLC) and high-performance liquid chromatography (HPLC) techniques have been suggested for analysis of the formulation.[7] HPLC is the most widely used technique for the estimation of lamivudine in human plasma, saliva, cerebrospinal fluid, and human blood cells, as well as for studying the drug metabolites in the urine.[8] The suggested HPTLC and HPLC methods for assay of lamivudine are expensive and need complex and sophisticated instrumentation.[8] Lamivudine can also be determined by Reverse Phase-HPLC method with lesser runtime, but the aforementioned drawback still persists.[9] One of the first methods for visible spectrophotometric determination of lamivudine was based on the colored condensation products of aromatic aldehydes;[10] this method suffers from a drawback as the interference from the excipients is more since the determination is carried out at much shorter wavelengths. It is also reported that lamivudine can also be assayed by titrimetric methods based on diazocoupling, redox reaction using Folin-Ciocalteu reagent, and redox-complexation reaction using ferric chloride-orthophenanthroline.[11] However, the above mentioned titrimetric methods are reported to suffer from disadvantages like unstablity of the reagents, high cost of the chemicals, reduced sensitivity, etc.[12] The present research work describes a UV spectrophotometric method for estimation of lamivudine in API and its pharmaceutical preparation.

## MATERIALS AND METHODS

Elico SL-159 UV-visible spectrophotometer equipped with a matched quartz cells ultrasonic bath was used to carry out the assay. The solvent used for the assay was spectroscopic-grade methanol.

### Evaluation of wavelength

**Figure 1:**
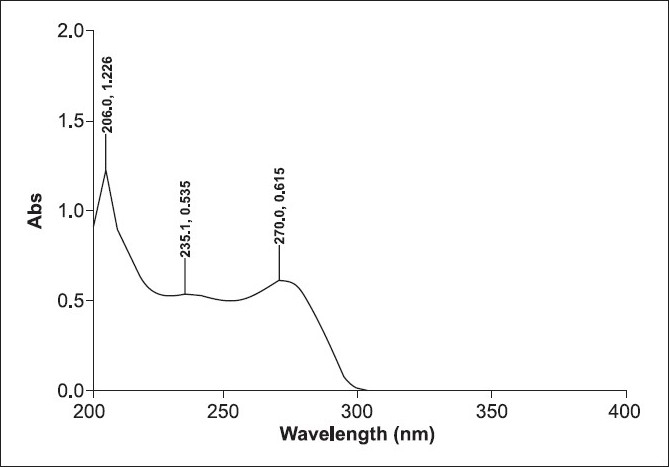
UV scan of lamivudine in methanol

About 10 µg/mL of lamivudine drug substance was accurately prepared in spectroscopic-grade methanol solvent. This preparation was then scanned in the 200—350 nm UV region. The wavelength maxima (λ_max_) was observed at 270 nm and this wavelength was adopted for absorbance measurement [Figure 1].

### Standard preparation

Accurately weighed 100 mg of lamivudine test standard was transferred to a volumetric flask containing 25 mL of methanol solvent. This was sonicated for about 5 min to dissolve it and the resultant solution was diluted to 100 mL with methanol solvent. Ten milliliters of this standard preparation was transferred to another volumetric flask and then diluted to 100 mL with methanol solvent.

### Sample preparation

Ten tablets from the marketed sample were weighed and crushed uniformly with the help of a mortar and pestle. An accurately weighed powder sample equivalent to 100 mg of lamivudine was transferred into a volumetric flask containing 25 mL methanol solvent. The contents were sonicated for about 5 min so that the dissolution is enhanced and is completed in 15 min. Ten millititers of the supernatant solution was then taken and diluted to 100 mL with methanol solvent [Table 1].

**Table 1:** Analysis data of tablet formulation

Sample	Label claim (mg/tablet)	Amount obtained (mg/tablet)	Percentage	Relative standard deviation (*n*=6)
Brand I	100	98.9	98.9	0.38%
Brand II	100	99.2	99.2	0.42%

### Procedure

The baseline correction on the UV spectrophotometer was performed using methanol as blank solvent in both reference and sample quartz cells. The aim was to obtain the absorbance of the standard (10 µg/mL of lamivudine) and sample preparations at 270 nm and determine the content of C_8_H_11_N_3_O_3_S in each tablet.

## VALIDATION

The method was validated for specificity, linearity, accuracy, ruggedness, and solution stability.

*Specificity*: The specificity of the method was established by measuring the interference, if any, observed due to the methanol solvent at the wavelength maxima of lamivudine. No significant absorbance due to methanol was observed at 270 nm [Figure 1].

### Linearity

The linearity of the method was established by determining the absorbance of different concentrations of lamivudine drug substance over a range of 50% (5 µg/mL) to 150% (15 µg/mL) of the normal sample preparation. Each level was measured in triplicate.

**Figure 2:**
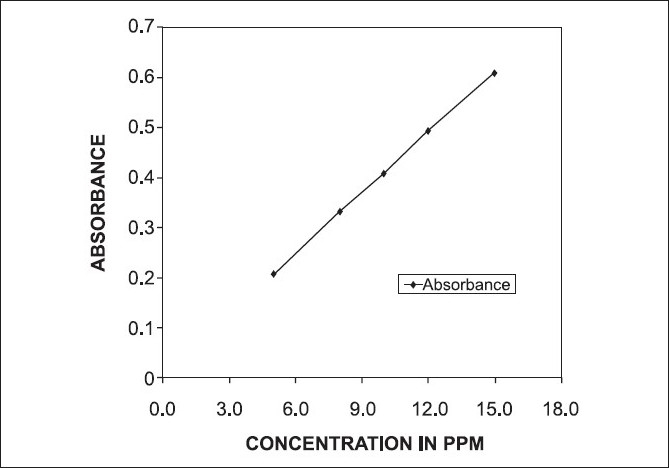
UV spectrophotometric determination of Lamivir^®^ 100 mg tablet. Linearity experiment

The calibration curve, as plot of absorbance vs concentration in µg/mL of lamivudine, was found to be rectilinear for 5, 8, 10, 12, and 15 µg/mL concentrations of lamivudine. The correlation coefficient was found to be more than 0.9998 [Figure 2].

### Precision

The assay of the same batch was performed in six replicates and the percentage relative standard deviation (%RSD) measured. The %RSD was found to be not more than 0.5%.

### Accuracy

The accuracy of the method was established by adding the lamivudine test standard solution of the pre-analyzed tablet formulation. The analysis at each level was performed in triplicate and the mean recovery of lamivudine was measured. The percent recovery at each level was found to be well within the range of 98.0%—102.0%, indicating insignificant interference from the excipients.

### Ruggedness

The ruggedness of the method was established by having the precision study performed on another instrument by another analyst. The cumulative %RSD for content of lamivudine for the samples of precision and ruggedness study were found to be not more than 1.0%.

### Solution stability

The absorbance of the same sample solution at the initial stage and intervals of 4 hours, 8 hours, 12 hours, and 24 hours were measured and the cumulative %RSD determined. The %RSD was found to be not more than 2.0%

## CONCLUSION

The proposed method for the assay of the popular anti-retroviral agent lamivudine in the commercially available tablet formulation is simple, accurate, economical, and rapid. It can be easily adopted for routine quality control for monitoring the assay in the API, in-process samples, and the finished tablet formulation. The method can be extended for studying the dissolution profile.
